# Dephosphorylation of juxtamembrane serines and threonines of the NPR2 guanylyl cyclase regulates oocyte meiotic resumption

**DOI:** 10.1186/2050-6511-16-S1-A30

**Published:** 2015-09-02

**Authors:** Leia C Shuhaibar, Aaron B Edmund, Jeremy R Egbert, Siu-Pok Yee, Lincoln R Potter, Laurinda A Jaffe

**Affiliations:** 1 Department of Cell Biology, University of Connecticut Health Center, Farmington, Connecticut, USA; 2 Department of Biochemistry, Molecular Biology and Biophysics, University of Minnesota, Minneapolis, Minnesota, USA; 3 Department of Genetics and Genome Sciences, University of Connecticut Health Center, 263 Farmington Avenue, Farmington, CT 06030, USA

## Background

**Figure 1:**
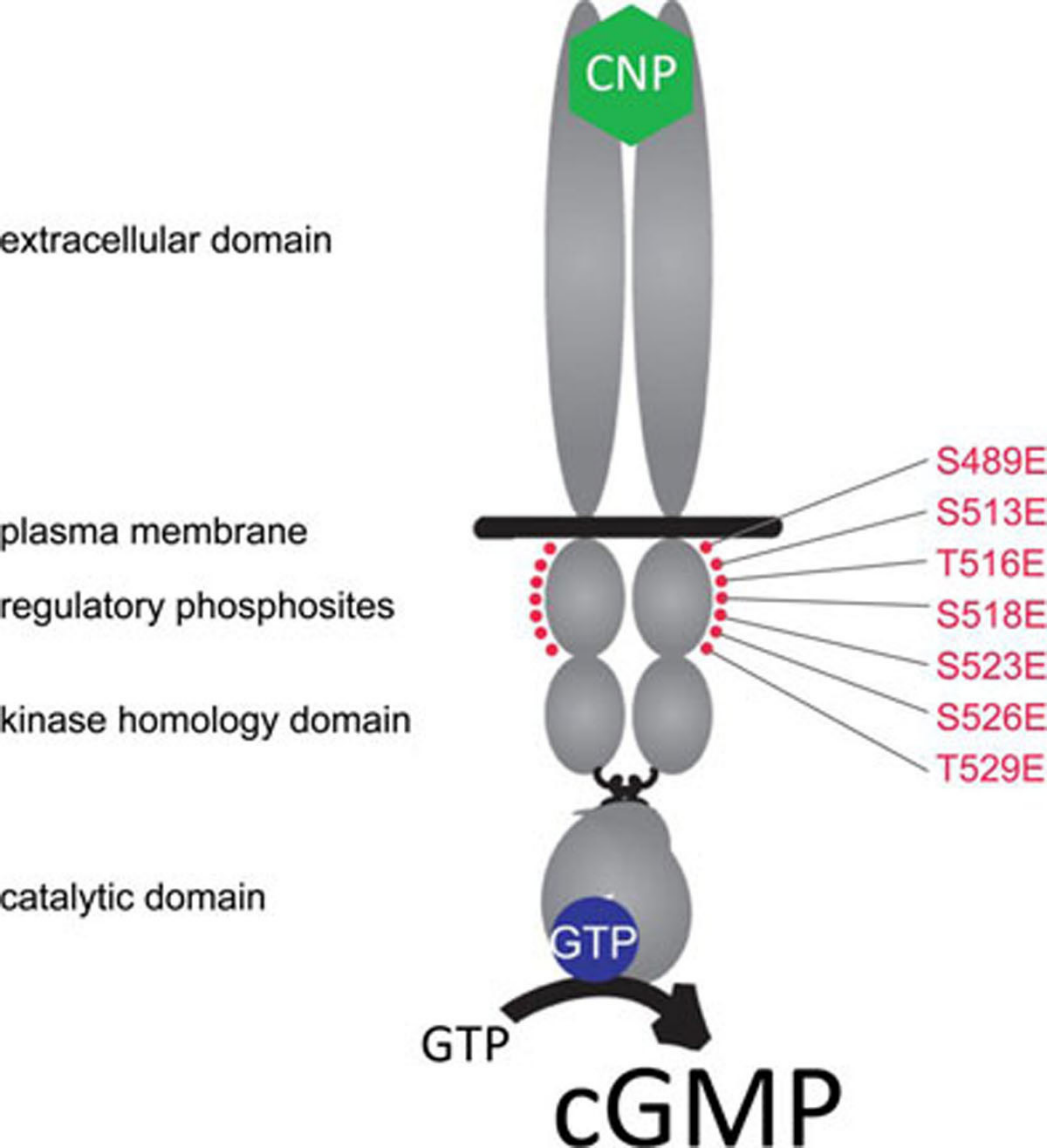
**Diagram of NPR2 showing the seven serine and threonine phosphorylation sites that were changed to glutamate (E) in the *Npr2*-7E mouse.** The functional domains of the homodimeric transmembrane protein NPR2 are shown in gray. Binding of CNP (green) to the extracellular domain and phosphorylation of seven serine and threonine sites (red) is required for CNP-dependent activation of the guanylyl cyclase activity of NPR2. Dephosphorylation of these regulatory sites results in a decrease in guanylyl cyclase activity; the 7E mutation results in a protein that cannot be dephosphorylated.

The meiotic cell cycle of mammalian oocytes starts during embryogenesis and then pauses until luteinizing hormone (LH) restarts the cycle. This meiotic arrest is maintained by cGMP, which is produced in the granulosa cells by C-type natriuretic peptide (CNP) activation of NPR2 [1]. LH decreases cGMP in the granulosa cells, and via equilibration through gap junctions, cyclic GMP also decreases in the oocyte, thus releasing the meiotic arrest [2]. LH causes dephosphorylation and inactivation of NPR2 [3,4], but whether NPR2 dephosphorylation is required for meiotic resumption is not known. Seven regulatory NPR2 phosphorylation sites have been identified (Fig. 1) [5,6]. Here, we generated a knock-in mouse where each site was mutated to glutamate (Npr2-7E), resulting in a “constitutively phosphorylated” enzyme that we used to investigate the role of NPR2 dephosphorylation in the rapid resumption of meiosis in response to LH.

## Results

**Figure 2:**
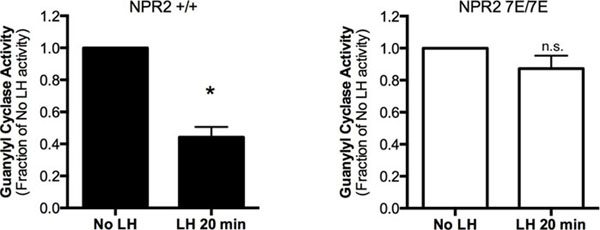
**Inhibition of the LH-induced decrease in guanylyl cyclase activity in mouse follicles by the *Npr2* -7E mutations.** Guanylyl cyclase activity in wild-type and Npr2-7E/7E follicle membranes, measured in the presence of 1 µM CNP. Membranes were prepared from follicles with or without treatment with LH for 20 min. Each value shows the mean ± s.e.m. for 4 separate membrane preparations. The asterisk indicates that LH treatment significantly decreased the CNP-dependent guanylyl cyclase activity in wild-type follicles (P<0.05). LH treatment did not cause a significant change in CNP-dependent activity in *Npr2*-7E/7E follicles (n.s.).

Membranes from isolated antral follicles treated with or without LH for 20 min were assayed for guanylyl cyclase activity under physiological conditions with CNP, ATP and Mg2+GTP (Fig. 2). In wild-type follicles treated with LH, the CNP-dependent guanylyl cyclase activity decreased to ~47% of initial values (Fig. 2, left). In contrast, LH caused no significant change in CNP-dependent guanylyl cyclase activity in Npr2-7E/7E follicle membranes (Fig. 2, right). Protein loss did not explain the reduction in CNP-dependent activity because activities measured with Mn2+GTP/Triton X-100 were not reduced by LH. These findings indicate that dephosphorylation of NPR2 is necessary for the hormonal regulation of guanylyl cyclase activity in the ovarian follicle.

To investigate whether the LH-induced decrease in guanylyl cyclase activity is required for meiotic resumption, we isolated Npr2-7E/7E and wild-type follicles and observed them in culture before and after addition of LH. In wild-type follicles, LH-induced nuclear envelope breakdown (NEBD) began at 2 hours and reached ~80% by 6 hours. In contrast, in Npr2-7E/7E follicles, no evidence of meiotic resumption was observed in the first 6 hours following treatment with LH. However, by 8 hours after LH application, NEBD had occurred in ~40% of Npr2-7E/7E follicle-enclosed oocytes, and by 12 hours NEBD was seen in ~80% of oocytes. During cumulus expansion preceding ovulation, gap junction communication between the oocyte and cumulus cells is disrupted [7]. The LH-induced cumulus expansion occurred similarly in Npr2-7E/7E and wild-type follicles, suggesting that gap junction disruption during cumulus expansion might explain why meiosis eventually resumes in the NPR2-7E/7E follicles.

## Conclusion

Dephosphorylation of NPR2 is required for the rapid resumption of meiosis in response to LH.
